# The reduction of hypoglycemia among inpatients through a multidisciplinary team work using a PDCA approach

**DOI:** 10.1186/1758-5996-7-S1-A48

**Published:** 2015-11-11

**Authors:** Thalita Barreira Modena Cardim, Magda Tiemi Yamamoto, Adriana de Fátima Avansi, Gustavo Daher, Rodrigo Bomeny de Paulo, Claudia Regina Laselva, Rogério Silicani Ribeiro, Jose Antonio Maluf de Carvalho

**Affiliations:** 1 Hospital Israelita Albert Einstein, São Paulo, Brazil

## Background

Hypoglycemia is associated with transient cognitive deficits and can result in cardiac arrhythmia, neurological damage, falls or aspiration. Among critical and non-critical inpatients, hypoglycemia increases the risk of death. Since 2012, we had kept the rate of hypoglycemia (proportion of capillary glucose episodes bellow 60 mg/dL per 100 glucose measurements) well controlled. During the first 3 months of 2014 we were aiming to keep the rate under 0,46%. However, there was an average of 149 episodes/month, which was equivalent to the rate of 0,57%.

## Objective

To improve the frequency of hypoglycemic episodes among inpatients.

## Materials and methods

Through a multidisciplinary team (pharmacists, nurses, physicians and nutritional therapists), using a PDCA approach (plan–do–check–act), we evaluated the main causes of hypoglycemia after 04/2014 and establish actions to improve the results.

## Results

**Figure 1:**
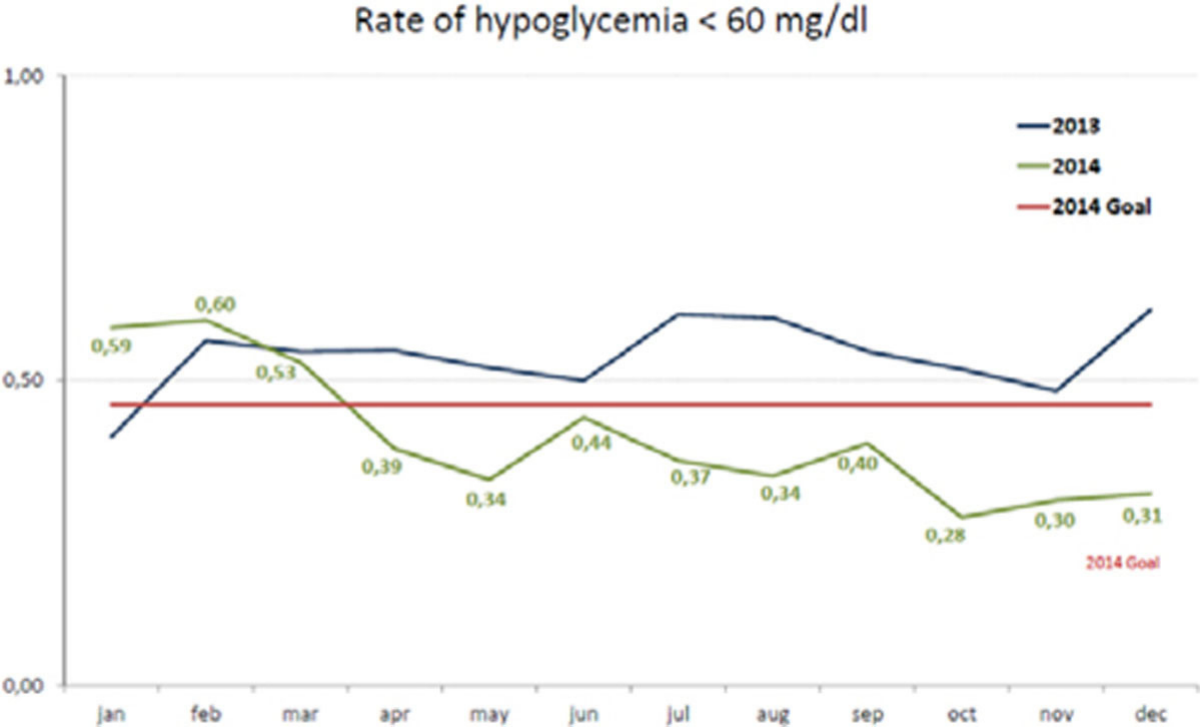
Rate of hypoglycemia < 60mg/dL

The most common causes were insufficient CHO intake, excessive glucose monitoring in terminally ill patients and inadequate insulin doses management. Consequently, we implemented protocols to guarantee a minimal intake of CHO (intravenous and/or oral), and discussions with the palliative care staff regarding the reduction of glycemic tests in terminally ill patients and improved the electronic prescription system. In addition, we developed pre-admission inquire to identify patients at risk of hypoglycemia before elective surgery and an electronic alarm to identify drugs associated with hypoglycemia. By these actions, the rate of hypoglycemia progressively reduced from 0,53% (139 episodes) in March/2014 to 0,31% (80 episodes) in December/2014, as observed in Figure 1.

## Conclusions

This project demonstrates the importance of integration and involvement of a multidisciplinary team and the importance of adaptation and creation of new standards for continuous process improvement to reduce the frequency of hypoglycemia among inpatients.

